# Salusin-β Not Salusin-α Promotes Vascular Inflammation in ApoE-Deficient Mice via the I-κBα/NF-κB Pathway

**DOI:** 10.1371/journal.pone.0091468

**Published:** 2014-03-12

**Authors:** Cheng-Hua Zhou, Lian Liu, Lu Liu, Ming-Xing Zhang, Hao Guo, Jin Pan, Xiao-Xing Yin, Teng-Fei Ma, Yu-Qing Wu

**Affiliations:** 1 School of Pharmacy, Xuzhou Medical College, Xuzhou, Jiangsu Province, China; 2 Jiangsu Province Key Laboratory of Anesthesiology, Xuzhou Medical College, Xuzhou, Jiangsu Province, China; 3 Department of Anesthetic Pharmacology, Xuzhou Medical College, Xuzhou, Jiangsu Province, China; VCU, United States of America

## Abstract

**Objective:**

Vascular inflammation plays an important role in the development and progression of atherosclerosis. Recently, salusins (salusin-α and salusin-β) have been reported to be associated wtih atherosclerosis. However, its underlying mechanism remains incompletely known. In this study, we observed the effects of salusins on vascular inflammation in apoE-deficient (apoE-/-) mice.

**Methods and Results:**

Six-week old male apoE-/- mice were infused with salusin-α, salusin-β or vehicle for 8 weeks via osmotic mini-pumps. Our results showed that apoE-/- mice receiving vehicle alone developed severe atherosclerotic lesions and dyslipidemia, with significantly up-regulated levels of IL-6, TNF-α, VCAM-1 and MCP-1. For apoE-/- mice receiving 8 weeks of salusin-β infusion, the atherosclerotic lesions were markedly aggravated, and the levels of IL-6, TNF-α, VCAM-1 and MCP-1 were substantially increased, despite a similar plasma lipid concentration with that of apoE-/- mice. However, after 8 week-infusion of salusin-α, apoE-/- mice presented significant amelioration in atherosclerotic lesions, along with remarkably up-regulated level of high-density lipoprotein-cholesterol (HDL-C) and down-regulated levels of IL-6 and TNF-α, but without any effect on the expressions of VCAM-1 and MCP-1. Furthermore, the activation of nuclear factor-κB (NF-κB), an important transcription factor essential for inflammatory molecules, and the degradation of I-κBα, an inhibitor of NF-κB, were markedly increased in apoE-/- mice receiving vehicle alone. Treatment with salusin-β not salusin-α could remarkably accelerate the process of NF-κB nuclear translocation and I-κBα degradation.

**Conclusion:**

Salusin-β, but not salusin-α, promotes vascular inflammation in apoE-deficient mice via the I-κBα/NF-κB pathway. These findings provide further insight into the mechanism of salusins in atherosclerosis and potential targets for the prevention and treatment of atherosclerosis.

## Introduction

Atherosclerosis is an important underlying pathology of cardiovascular diseases, the leading cause of death in developed countries with increasing incidence trends in developing countries [1], [2]. Recently, the pathogenesis of atherosclerosis is widely attributed to vascular inflammation [3]. An increasing number of studies have demonstrated the crucial role of inflammation in each stage of atherosclerosis ranging from initiation through progression to the formation of thrombotic complications. Moreover, vascular endothelial dysfunction induced by hyperlipidemia, hypertension, free radicals, diabetes, infection, shear stress and other factors is regarded as one of the first steps toward plaque formation [4].

Salusins is a new class of vasoactive peptides originally identified by Shichiri et al. in 2003, which includes salusin-α and salusin-β consisting of 28 and 20 amino acids respectively [5]. Salusins are expressed in human coronary atherosclerotic plaques, with lower expression of salusin-α than salusin-β [6], suggesting the involvement of salusins in the development and progression of atherosclerosis. Further studies show that salusin-α can suppress human foam cell formation by down-regulation of acyl-coenzyme A: cholesterol acyltransferase-1 (ACAT-1), stimulating the accumulation of cholesterol ester in macrophages. In contrast, salusin-β results in an increase in the formation of human foam cells by up-regulating ACAT-1 [6]. Moreover, compared with healthy volunteers, patients with coronary artery diseases manifest reduced concentrations of serum salusin-α [6]. Serum salusin-α level is also associated with atherosclerosis and left ventricular diastolic dysfunction in essential hypertension [7], [8]. In addition, our recent study shows that the expression of salusin-β is increased in the vascular tissues of low-density lipoprotein receptor-deficient (LDLR-/-) mice fed on high-fat diets compared with that of C57BL/6 control mice [9]. These investigations indicate that salusin-α is likely to prevent atherosclerosis, while salusin-β may act as a potential proatherogenic factor. Indeed, Nagashima et al. has demonstrated that chronic salusin-β infusion into apolipoprotein E-deficient (apoE-/-) mice can accelerate the development of early atherosclerotic lesions, while chronic salusin-α infusion blocks the progression of atherosclerotic lesion [10]. Our previous study also shows that subcutaneous injection of salusin-β can significantly aggravate the atherosclerotic lesions in LDLR-/- mice [9].

Despite the clear effects of salusin-α and salusin-β on atherosclerosis, further studies are needed to explore their underlying mechanisms. Sato et al. finds that salusin-β can be released from THP-1 and U937 human monoblastic leukemia cell lines, while stimulation of THP-1 and U937 by the inflammatory cytokines, tumor necrosis factor-α (TNF-α) and lipopolysaccharide (LPS), results in increased secretion of salusin-β [11], suggesting a potential relationship between salusin-β and inflammatory reaction. Due to the above potential relationship and the crucial role of inflammation in the development and progression of atherosclerosis, we assume that salusins may regulate atherosclerosis by modulating inflammatory responses. In fact, Koya et al. has indicated that salusin-β can accelerate inflammatory responses in vascular endothelial cells via nuclear factor-κB (NF-κB) signaling [12]. However, according to Koya's report, the effect of salusin-β is indirectly observed through infusion of antiserum against salusin-β in vivo, while the effect of salusin-α has not studied yet. Therefore, the present study is to investigate the effects of salusin-α and salusin-β on the vascular inflammation of atherosclerosis through continuous infusion of salusins into ApoE-/- mice.

## Materials and Methods

### Animals

Animal care and experimental procedures were approved and performed in accordance with the Institutional Animal Care and Use Committee of Xuzhou Medical College. Six-week old male C57BL/6 wild-type mice and apoE-/- mice (with C57BL/6 background) were purchased from the Department of Laboratory Animal Science, Peking University Health Science Center. Mice were housed in a temperature-controlled environment (25°C) with 12/12- hour light/dark cycles and given free access to food and water. After two weeks of acclimatization, apoE-/- mice were randomly divided into an apoE-/- group, a salusin-α group and a salusin-β group. C57BL/6 wild-type mice were used as control. All the mice were fed a high-fat diet containing 0.15% (w/w) cholesterol and 21% (w/w) fat (the Animal Center of Xuzhou Medical College, Xuzhou, China) for 8 weeks to induce the development of atherosclerosis.

### Continuous infusion of salusins into mice

Salusin-α and salusin-β dissolved in saline were continuously infused into apoE-/- mice at a rate of 0.6 nmol/kg/h for 8 weeks via ALZET osmotic mini-pumps (Model 1002, Cupertino, CA) as previously described [13]. Briefly, mice were anesthetized with diethyl ether until failing to respond to tactile stimulation. A small incision was made on the dorsum, slightly posterior to the scapulae, where a subcutaneous pocket was created by blunt dissection. Then, a filled ALZET pump was inserted into the pocket, with delivery portal first. The mice in the control group and apoE-/- group were received saline vehicle alone.

### Analysis of plasma lipids

After an overnight fast, blood samples were collected by exsanguination from the retroorbital venous plexus of mice. The concentrations of plasma total cholesterol (TC), triglycerides (TG), low-density lipoprotein-cholesterol (LDL-C) and high-density lipoprotein-cholesterol (HDL-C) were determined enzymatically using commercially available kits (Nanjing Jiancheng Bioengineering Institute, Nanjing, China).

### Evaluation of atherosclerotic lesions

The heart and proximal aorta were removed from the mice and fixed using 4% paraformaldehyde. After snap-frozen in OCT compound, 10 μm-thick serial sections were cut from the proximal 1 mm of the aortic root. Aortic root cross-sections were stained with Oil red O and counterstained with hematoxylin. The Oil red O stained areas were quantified using Image J Software. Data were expressed as the percentage of total intimal area with positive Oil red O staining. The above frozen sections were also stained with hematoxylin and eosin (H&E) to observe the atherosclerotic lesions. Samples were examined under a microscope blindly by two experienced pathologists to examine the presence of fatty streak, fibrous plaque and atheroma.

### Enzyme-linked immunosorbent assay (ELISA) for plasma TNF-α and IL-6 levels

Plasma levels of TNF-α and interleukin-6 (IL-6) were measured with ELISA kits according to the manufacturers' instructions (Shanghai Westang Biotechnology Co. Ltd, Shanghai, China). Briefly, 100 μl of the blank, standards and treated samples (10-fold dilution with sample buffer) were added to appropriate coated wells in 96-well plates. The plates were fully blended and incubated at 37°C for 2 hours. After the addition of biotinylated antibody (100 μl), the plates were incubated at 37°C for another one hour. Then the wells were washed with washing buffer followed by the addition of the HRP-conjugated antibody. Furthermore, the plates were incubated at 37°C for 30 minutes and fully washed. Also, HRP substrate 3, 3, 5, 5-tetramethylbenzidine solution was added to the plate and incubated at 37°C for 15 minutes in darkness. The reaction was terminated with stopping solution (100 μl). Finally, the yellow color developed was read at 450 nm using a microplate reader (128ce, Clinibio).

### Immunohistochemistry for VCAM-1, MCP-1 and NF-κB p65 protein expressions

The aortic arch was embedded in paraffin and cut into 4 μm thick sections. Immunostainning was performed as previously reported [9]. Briefly, paraffin sections were stained with rabbit anti-mouse antibodies against vascular cell adhesion molecule-1 (VCAM-1, 1:200, Santa Cruz Biotechnology Inc, CA, USA), monocyte chemoattractant protein-1 (MCP-1, 1:100, Boster Biological Technology, Ltd., Wuhan, China) and NF-κB p65 (1:1000, Abcam, Cambridge, MA) at 4°C overnight, followed by HRP-coupled anti-rabbit IgG as a secondary antibody. Then the wells were incubated with streptavidin-biotin-peroxidase complex (SABC). The activity of peroxidase was determined through reaction with 3, 3′-diaminobenzidine (DAB) reagent, and the sections were counterstained with hematoxylin. Negative controls were performed using PBS instead of primary antibodies. The mean optical densities of immunostaining for VCAM-1 and MCP-1 were quantified using Image-Pro Plus Analysis Software, while the nuclear positive rate of NF-κB was counted.

### Real-time quantitative polymerase chain reaction for VCAM-1 and MCP-1 mRNA expressions

The abdominal aortas isolated from mice were fresh-frozen in liquid nitrogen and then stored at −80°C. Total RNA was extracted using Trizol reagent (Invitrogen, USA) according to the manufacturer's instructions. Then High-Capacity cDNA Reverse Transcription Kit (Applied Biosystems, Foster City, USA) was used for reverse transcription of total mRNA to cDNA. The PCR primers used were as follows: MCP-1:5′-GGT CCC TGT CAT GCT TCT GG-3′ (forward), and 5′-CCT GCT GCT GGT GAT CCT CT-3′ (reverse); VCAM-1: 5′-GAA CCC AAA CAG AGG CAG AG-3′ (forward), and 5′-GGT ATC CCA TCA CTT GAG CAG-3′ (reverse); β-actin: 5′-GAG ACC TTC ACC CCA GC-3′ (forward), and 5′-ATG TCA CGC ACG ATT TCC C (reverse). In this assay, approximate 1.5 μg of total RNA samples were added to a thermal cycler using the following conditions: hot-start activation for 2 minutes at 95°C, followed by 40 cycles of 95°C for 15 seconds and 60°C for 60 seconds. Each cycle ends with a single fluorescent reading and the program was completed with a melting curve analysis. Real-time PCR analysis was performed through Roche 480 LightCycler® detection system using GoTaq® qPCR Master Mix Kit (Promega, Madison, WI, USA). Each sample was analyzed in triplicate. The relative amount of target mRNA was normalized to the reference, and calculated by the comparative crossing point (Cp) method. The specificity of the amplified product was monitored by its melting curve.

### Western blotting analysis for VCAM-1 and I-κBα protein expressions

The thoracic aortas were excised and homogenized in lysis buffer containing 50 mM Tris (pH 7.4), 150 mM NaCl, 1% Triton X-100, 1% sodium deoxycholate and 0.1% sodium dodecyl sulfate (SDS). After centrifugation at 12,000 g for 10 minutes, the resulting supernatant was processed to yield total protein samples. The protein concentrations were determined by the BCA Protein Assay Kit (Beyotime institute of Biotechnology, China) according to the manufacturer's instructions. Equal amounts of protein samples were boiled for 10 minutes in SDS-PAGE Sample Loading Buffer (Beyotime institute of Biotechnology, China) and loaded onto a 10% SDS-polyacrylamide gel before running. Then the proteins were transferred from the gel to 0.2 μm polyvinylidene fluoride (PVDF) membrane and blocked at room temperature for 2 hours. The membrane was incubated at 4°C overnight with primary antibodies including anti-VCAM-1 (1:200, Santa Cruz Biotechnology) and anti-inhibitory κBα (I-κBα, 1:2000, Abcam) followed by secondary antibody conjugated to alkaline phosphatase (1:1000, Zhongshan Golden Bridge Biotechnology Co., Beijing, China) at room temperature for 2 hours. Blots were developed using a BCIP-NBT kit (Promega, Madison, WI, USA). All blots were stripped and reprobed with polyclonal anti-β-actin to verify equal protein loading.

### Statistical analysis

All the results were shown as the mean ± SD. Statistical differences of the experimental data were assessed by the one-way ANOVA followed by the Student-Newman-Keuls test. A value of *P*<0.05 was considered statistically significant.

## Results

### Effects of salusins on the progression of atherosclerotic lesions

**Figure 1:**
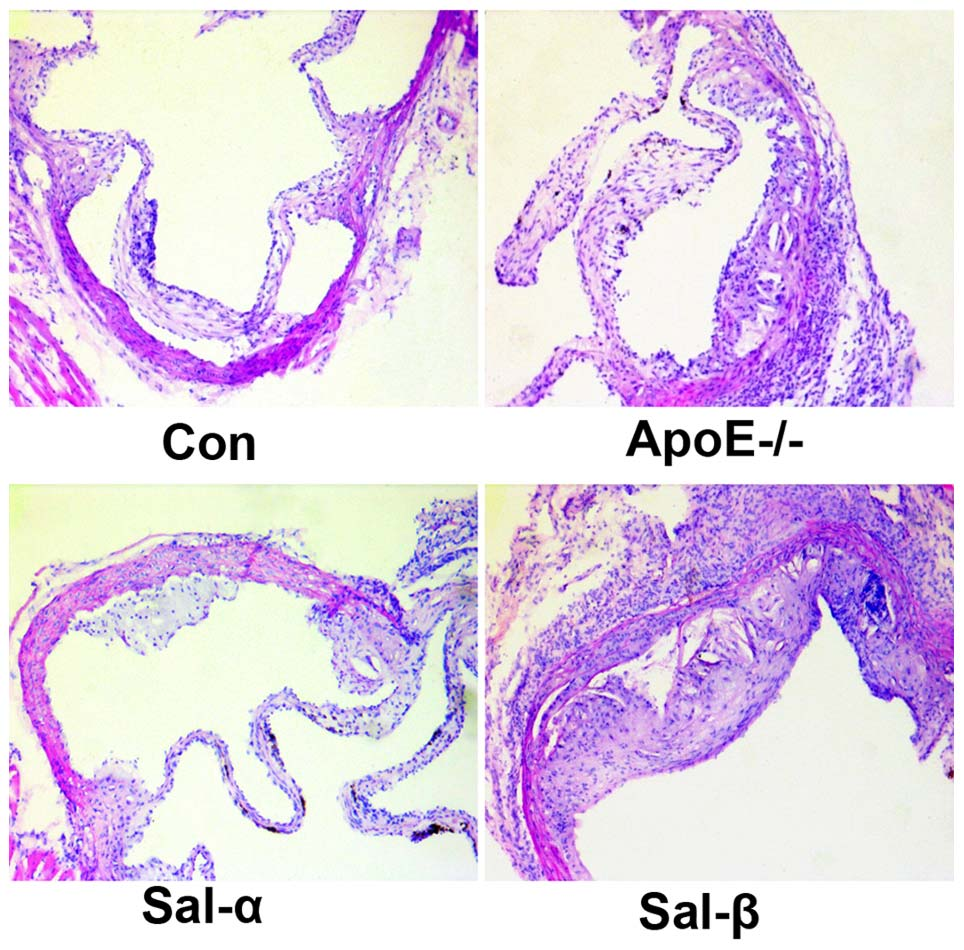
Hematoxylin and eosin (H&E) staining for histological analysis of atherosclerotic lesions. Salusin-α or salusin-β was continuously infused into apoE-/- mice for 8 weeks via osmotic mini-pumps. Frozen sections from the aortic root were stained with H&E. Representative photomicrographs were shown (magnification×100). Con: the C57BL/6 control mice; ApoE-/-: the vehicle-treated apoE-/- mice; Sal-α: the salusin-α-treated apoE-/- mice; Sal-β: the salusin-β-treated apoE-/- mice.

To ascertain the effects of salusins on the progression of atherosclerotic lesions, the histological changes in vascular tissues were observed through H&E staining. As shown in Figure 1, no atherosclerotic lesion was seen in C57BL/6 mice. ApoE-/- mice receiving vehicle developed atherosclerotic plaques with a small amount of cholesterol crystal, intimal thickening and fibrous cap in the aortic root within 8 week of high fat and cholesterol diets. ApoE-/- mice treated with salusin-β presented extensive aortic intimal thickening, and increased volume of atherosclerotic plaque including a large amount of cholesterol crystal. In contrast, infusion of salusin-α into apoE-/- mice could reduce the degree of atherosclerotic lesions where a small amount of fibrous plaques and intimal thickening were detected.

**Figure 2:**
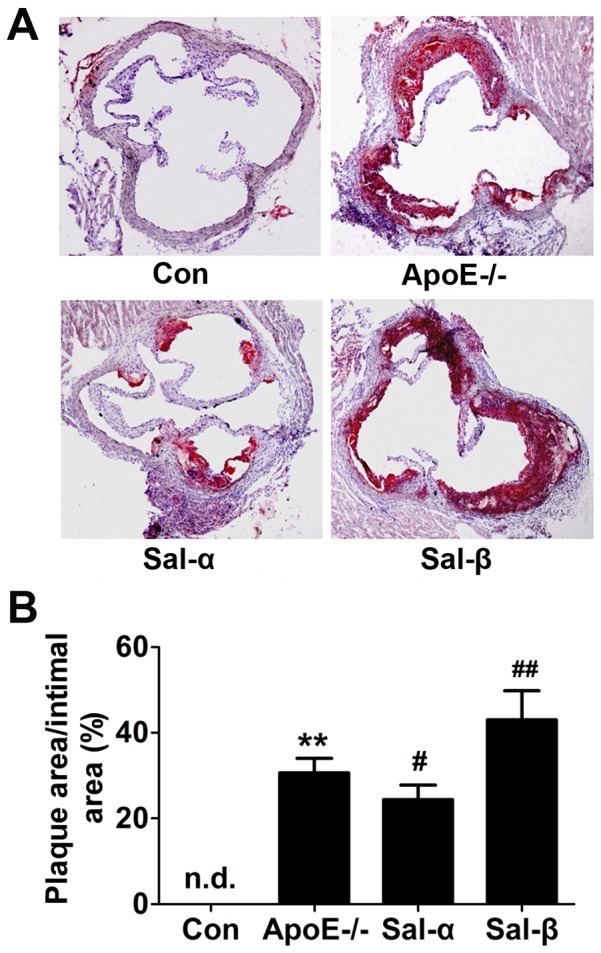
Oil red O staining for the formation of lipid-rich plaques. Salusin-α or salusin-β was continuously infused into apoE-/- mice for 8 weeks via osmotic mini-pumps. Frozen sections from the aortic root were stained with Oil red O. (A) Representative photomicrographs showing lipid-rich plaques in the aortic root of mice (magnification×40). (B) Quantification of lipid-stained areas in the aortic root by the ratio of the Oil red O-stained area to the total intimal area. Con: the C57BL/6 control mice; ApoE-/-: the vehicle-treated apoE-/- mice; Sal-α: the salusin-α-treated apoE-/- mice; Sal-β: the salusin-β-treated apoE-/- mice. Data were expressed as mean±SD (*n* = 4-6). **P<0.01 compared with the control group; ^#^ *P*<0.05, ^##^ *P*<0.01 compared with the apoE-/- group. “n.d.” indicates not detected.

In addition, the development of lipid-rich plaques was also evaluated by Oil red O staining. As shown in Figure 2A, lipid-rich plaques were clearly observed in vehicle-treated apoE-/- mice, which was absent in the control mice. In contrast, continuous infusion of salusin-β induced more extensive lipid-rich plaque formation, while salusin-α treatment could inhibit the development of lipid-rich plaques. Morphometric analysis revealed that the ratio of the plaque area to the intimal area was significantly increased in apoE-/- mice than that in the control mice (Figure 2B). The ratio was increased by 40.5% after salusin-β infusion (*P*<0.01), in comparison with 20.3% reduction after salusin-α exposure (*P*<0.05).

### Effects of salusins on the levels of plasma lipids

**Figure 3:**
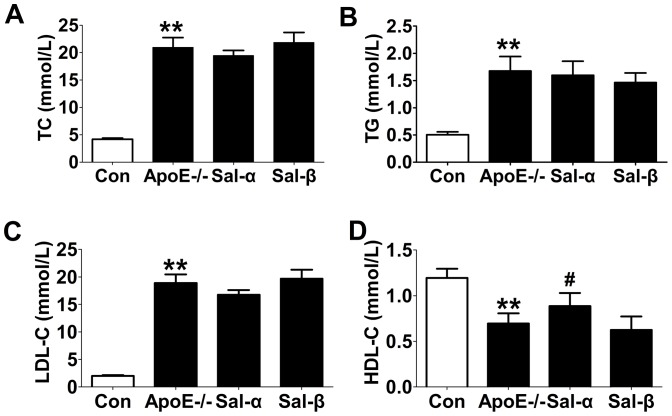
Effects of salusins on the levels of plasma lipids in mice. After 8 weeks of infusion with salusin-α or salusin-β, fasting blood samples were collected by exsanguination from the retroorbital venous plexus of mice. The concentrations of plasma total cholesterol (TC), triglycerides (TG), low-density lipoprotein-cholesterol (LDL-C), and high-density lipoprotein-cholesterol (HDL-C) were determined enzymatically using a commercially available kit. Con: the C57BL/6 control mice; ApoE-/-: the vehicle-treated apoE-/- mice; Sal-α: the salusin-α-treated apoE-/- mice; Sal-β: the salusin-β-treated apoE-/- mice. Data were expressed as mean±SD (*n* = 6). **P<0.01 compared with the control group; ^#^ *P*<0.05 compared with the apoE-/- group.

As shown in Figure 3, vehicle-treated apoE-/- mice presented higher plasma levels of TC, TG and LDL-C but a lower concentration of HDL-C than C57BL/6 control mice (*P*<0.01). Salusin-α infusion could significantly increase the plasma HDL-C level by 27.1% (*P*<0.05). No significant difference was observed in other lipid profiles.

### Effects of salusins on the levels of TNF-α and IL-6

**Figure 4:**
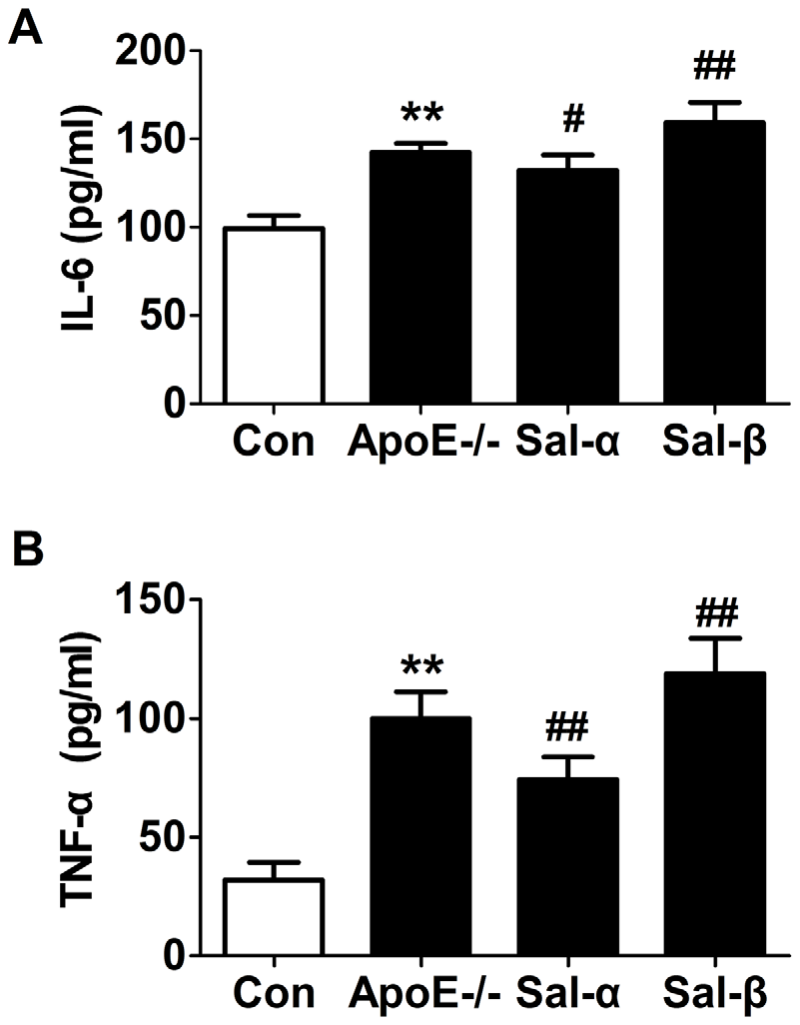
Effects of salusins on the plasma levels of IL-6 and TNF-α. After 8 weeks of infusion with salusin-α or salusin-β, fasting blood samples of mice were collected. The plasma levels of IL-6 and TNF-α were determined by enzyme-linked immunosorbent assay (ELISA). Con: the C57BL/6 control mice; ApoE-/-: the vehicle-treated apoE-/- mice; Sal-α: the salusin-α-treated apoE-/- mice; Sal-β: the salusin-β-treated apoE-/- mice. The results were presented as mean±SD (*n* = 6). **P<0.01 compared with the control group, ^#^ *P*<0.05, ^##^ *P*<0.01 compared with the apoE-/- group.

Salusins are considered a group of novel atherosclerosis targets related to inflammatory responses. Accordingly, we examined the plasma levels of two typical inflammatory cytokines TNF-α and IL-6 using an ELISA method. As shown in Figure 4, the plasma levels of TNF-α and IL-6 were apparently increased in vehicle-treated apoE-/- mice compared with those in C57BL/6 control mice (*P*<0.01). However, infusion of salusin-β into apoE-/- mice resulted in an obvious increase in the levels of TNF-α and IL-6 (*P*<0.01), while after salusin-α exposure the levels of TNF-α and IL-6 were reduced significantly (*P*<0.01, *P*<0.05).

### Effects of salusins on the expressions of VCAM-1 and MCP-1 mRNA

**Figure 5:**
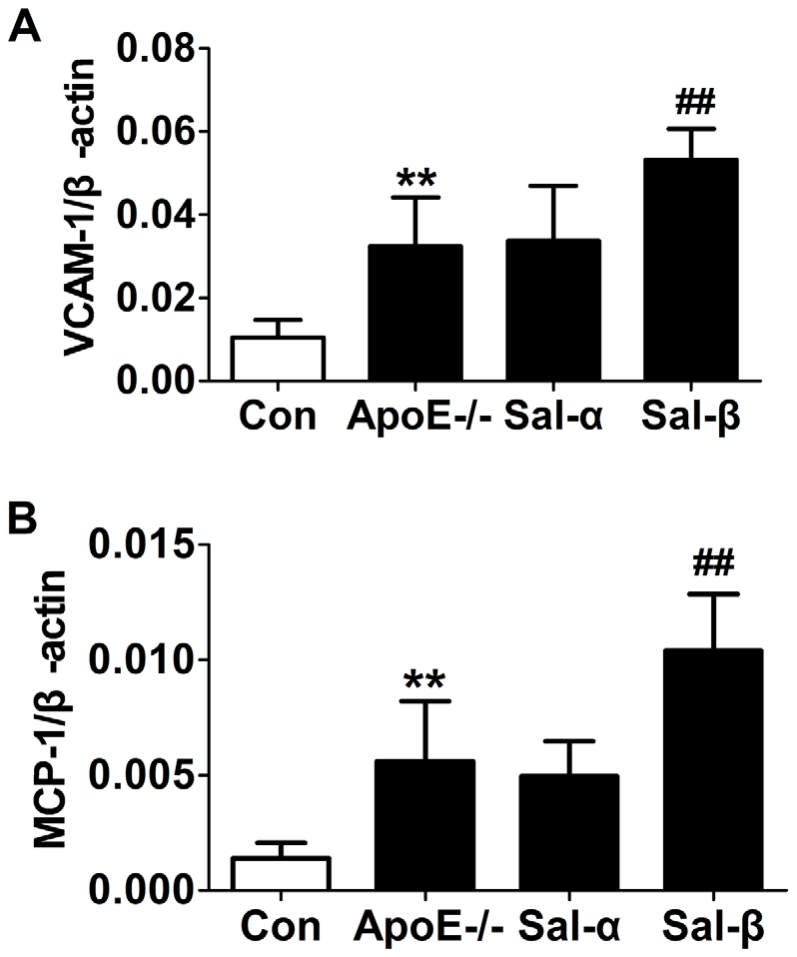
Effects of salusins on the expressions of VCAM-1 and MCP-1 mRNA in the aorta of mice. After 8 weeks of infusion with salusin-α or salusin-β, total RNA was extracted from the abdominal aortas of mice. The amounts of VCAM-1 and MCP-1 mRNA were then determined by Real-time quantitative PCR analysis. Con: the C57BL/6 control mice; ApoE-/-: the vehicle-treated apoE-/- mice; Sal-α: the salusin-α-treated apoE-/- mice; Sal-β: the salusin-β-treated apoE-/- mice. Data were normalized to the abundance of mRNA encoding β-actin, and expressed as mean±SD (*n* = 6). **P<0.01 compared with the control group; ^##^ *P*<0.01 compared with the apoE-/- group.

To observe the effects of salusins on vascular inflammation, the expressions of adhesion molecule VCAM-1 and chemokine MCP-1 at mRNA level within the aorta were examined by real-time quantitative PCR. As shown in Figure 5, vehicle-treated apoE-/- mice showed higher levels of VCAM-1 and MCP-1 mRNA than C57BL/6 control mice (*P*<0.01). Meanwhile, no significant difference was found for the expressions of VCAM-1 and MCP-1 mRNA between vehicle- and salusin-α treated apoE-/- mice. However, the mRNA levels of both VCAM-1and MCP-1 were significantly higher in apoE-/- mice treated with salusin-β than those treated with vehicle (*P*<0.01).

### Effects of salusins on the protein expressions of VCAM-1 and MCP-1

**Figure 6:**
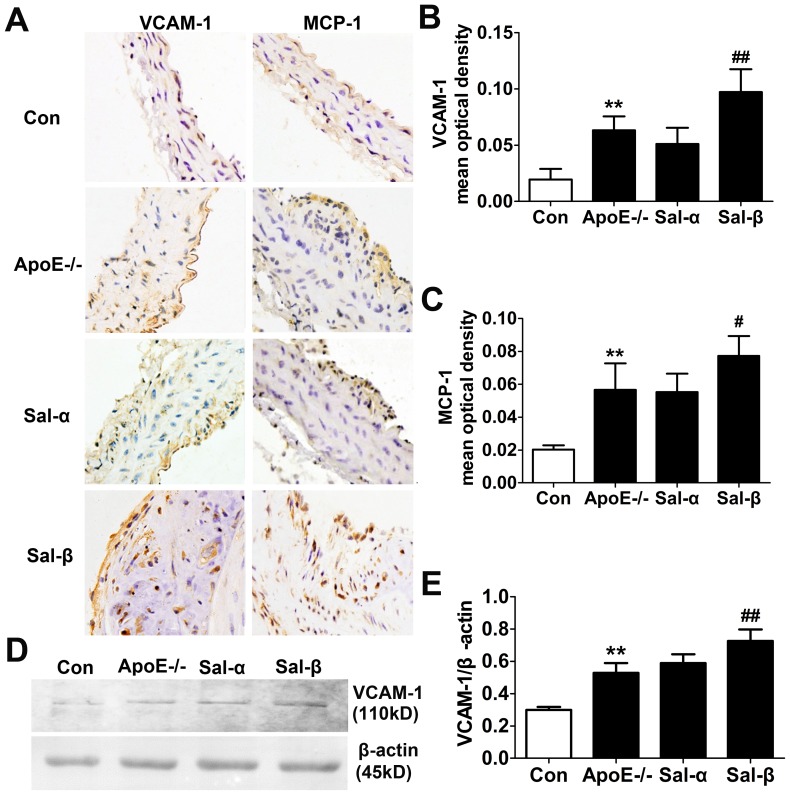
Effects of salusins on the protein expressions of VCAM-1 and MCP-1 in the aorta of mice. (A) Representative photomicrographs showing immunohistochemical staining for VCAM-1 and MCP-1. Immunoreactivity appears as a result of 3, 3′-diaminobenzidine (DAB) colorimetric reaction. (B) The mean optical densities of VCAM-1 immunostaining. (C) The mean optical densities of MCP-1 immunostaining. (D) Western blotting analysis for VCAM-1 protein expression. (E) Optical density analysis shown as the ratio of VCAM-1 to β-actin. Con: the C57BL/6 control mice; ApoE-/-: the vehicle-treated apoE-/- mice; Sal-α: the salusin-α-treated apoE-/- mice; Sal-β: the salusin-β-treated apoE-/- mice. The results were presented as mean±SD (*n* = 4-5). **P<0.01 compared with the control group, ^#^ *P*<0.05, ^##^ *P*<0.01 compared with the apoE-/- group.

In order to determine the immunolocalization of VCAM-1 and MCP-1 in the aortic arch, immunohistochemical staining was performed. As shown in Figure 6A-C, little VCAM-1 and MCP-1 immunostaining was found within the tissues of C57BL/6 control mice. Compared with the control mice, the amounts of VCAM-1 and MCP-1 proteins in vehicle-treated apoE-/- mice were prominent at the plaque lesions in the intimal side (*P*<0.01), which were enhanced by salusin-β treatment (*P*<0.01, *P*<0.05). The positive staining of VCAM-1 and MCP-1 in the salusin-α group was weaker than that in the apoE-/- group, while without statistical significance (*P>*0.05).

Western blotting analysis for VCAM-1 protein expression was shown in Figure 6D and E. The protein expression of VCAM-1 in vehicle-treated apoE-/- mice was significantly higher than that in C57BL/6 mice (*P*<0.01). Compared with apoE-/- mice treated with vehicle, the mice exposed to salusin-β presented increased expression of VCAM-1 protein (*P*<0.01), but no significant change was observed in salusin-α-treated mice (*P*>0.05). These results were consistent with the changes in mRNA level analyzed by real-time quantitative PCR.

### Effects of salusins on the protein expressions of NF-κBp65 and I-κBα

**Figure 7:**
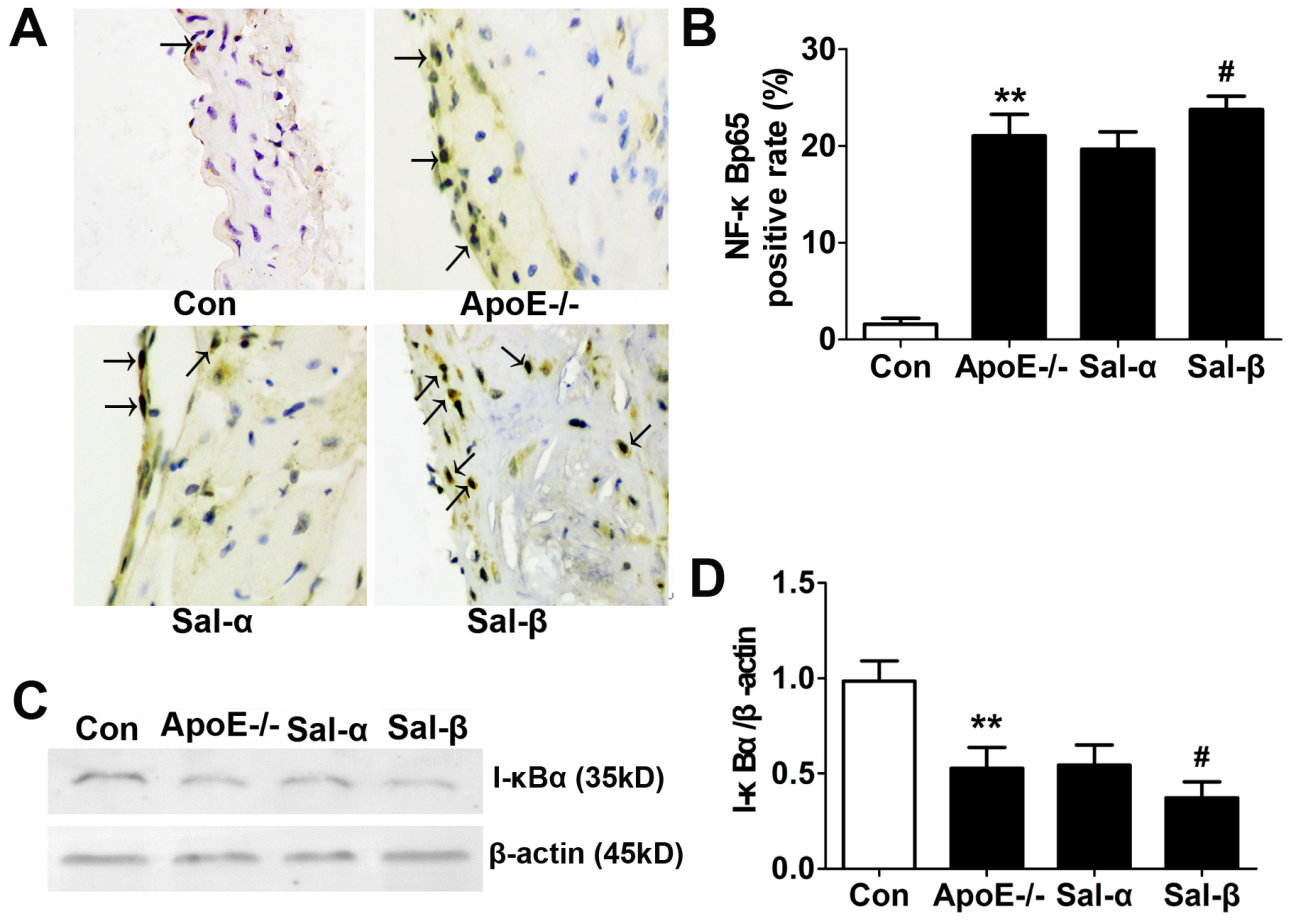
Effects of salusins on the protein expressions of NF-κB and I-κBα in the aorta of mice. (A) Representative photomicrographs showing immunohistochemical staining for NF-κB. Immunopositive cells (arrow) appear brown in the nuclei as a result of 3, 3′-diaminobenzidine (DAB) colorimetric reaction. (B) The percentage of nuclear positive cells was counted. (C) Western blotting analysis for I-κBα protein expression. (D) Optical density analysis shown as the ratio of I-κBα to β-actin. Con: the C57BL/6 control mice; ApoE-/-: the vehicle-treated apoE-/- mice; Sal-α: the salusin-α-treated apoE-/- mice; Sal-β: the salusin-β-treated apoE-/- mice. The results were presented as mean±SD (*n* = 5). **P<0.01 compared with the control group, ^#^ *P*<0.05 compared with the apoE-/- group.

As a key regulator of atherosclerosis, NF-κB regulates the expressions of various inflammatory factors, including VCAM-1, MCP-1, IL-6 and TNF-α, which in turn accelerate the progress of chronic inflammation. To further confirm the regulatory role of NF-κB transcription factor in inflammatory factor expression, I-κBα degradation in the cytoplasm was assessed by western blotting while NF-κBp65 activation in the nuclei was observed through immunohistochemistry. As shown in Figure 7, compared with C57BL/6 control mice, the nuclear positive percentage of NF-κBp65 was remarkably increased in apoE-/- mice (*P*<0.01), while the expression of I-κBα in the cytoplasm extract was markedly decreased (*P*<0.01). Compared with apoE-/- mice receiving vehicle alone, infusion of salusin-β into apoE-/- mice could obviously raise the nuclear positive percentage of NF-κBp65 (*P*<0.05) but remarkably reduce the protein expression of I-κBα (*P*<0.05). However, exposure to salusin-α could not result in any significant effect on the nuclear positive percentage of NF-κBp65 and the expression of I-κBα in the cytoplasm (*P*>0.05). These results indicated that salusin-β could promote I-κBα degradation, increasing NF-κB activation and p65 translocation into the nuclei.

## Discussion

Although the promoting effect of salusin-β on vascular inflammation is determined through infusion of antiserum against salusin-β [12], the effects of exogenous salusins on the vascular inflammation in *in vivo* atherosclerosis models remained incompletely known. Our current findings demonstrated that exogenous salusin-α could ameliorate the progression of atherosclerosis, which was related to an increased level of HDL-C while independent of vascular inflammation. In contrast, exogenous salusin-β could significantly aggravate the progression of atherosclerosis through stimulation of inflammatory markers.

To explore the effects of salusins on vascular inflammation in the development of atherosclerosis, we first assessed atherosclerotic lesions and lipid deposits after continuous infusion of exogenous salusins. In our present experiments, the atherosclerotic lesions in apoE-/- mice were ameliorated after 8-week infusion of exogenous salusin-α but aggravated when exogenous salusin-β was given. However, the result about salusin-β infusion was inconsistent with the earlier study, which demonstrating that chronic 8-week infusion of salusin-β has no effect on the progression of atherosclerosis in apoE-/- mice [10]. The discrepancy may be due to different ages of experimental mice adopted, which causes the development of atherosclerosis at various stages.

Furthermore, no significant difference was found for the levels of plasma TC, TG and LDL-C between the vehicle-treated apoE-/- mice and the salusin-α-treated mice, despite a higher concentration of HDL-C in salusin-α-treated mice than that in apoE-/- mice. Similarly, no significant difference was observed in plasma lipid levels after salusin-β infusion. Our data suggested that the anti-atherosclerotic role of salusin-α may be associated with the elevated level of HDL-C, which consistent with the previous report [10]. In addition, salusin-α has been reported to inhibit foam cell formation [6], [10], which may be one another mechanism for salusin-α to alleviate atherosclerosis.

Atherosclerosis is a complex and multifactorial disease, whose pathogenesis is associated with inflammatory responses. During the progression of atherosclerosis, adhesion molecules like VCAM-1 can promote monocyte adhesion to the intimal surface [14], [15]. MCP-1 is related to the migration of monocytes into the intima and accumulates in the injured regions in various vascular diseases, such as atherosclerosis [16], [17]. Subintimal monocytes convert into macrophages, which ingest lipids and then become foam cells. These cells and other arterial wall cells can release pro-inflammatory cytokines like IL-6 and TNF-α [4]. In the current research, the levels of IL-6, TNF-α, MCP-1 and VCAM-1 in apoE-/- mice were significantly increased. Moreover, increases in cytokine levels in patients with atherosclerosis were reported [18]. These findings indicate the presence of an active vascular inflammatory response in atherosclerosis. Meanwhile, the expressions of IL-6, TNF-α, MCP-1 and VCAM-1 were up-regulated in apoE-/- mice treated with salusin-β, indicating that salusin-β could aggravate the progression of atherosclerosis via up-regulation of inflammatory molecules. This result is consistent with other studies where salusin-β is reported to directly up-regulate the level of VCAM-1 in endothelial cells [12]. In contrast, salusin-α could selectively down-regulate the levels of IL-6 and TNF-α in plasma not MCP-1 and VCAM-1 in the aorta, suggesting little effects of salusin-α on inflammation in atherosclerosis progression.

Many genes encoding cytokines, chemokines and adhesion molecules including IL-6, TNF-α, MCP-1 and VCAM-1 are regulated by NF-κB, and greatly contribute to inflammatory responses [19]. Moreover, the generation and release of cytokines, including TNF-α, can further activate NF-κB and create a positive feedback, resulting in inflammatory signal amplification [20]. Activated NF-κB is present in the atherosclerotic lesions of apoE-deficient mice [21]. In our current study, NF-κB activation and I-κBα degradation were remarkably aggregated in apoE-/- mice. Exposure to salusin-β could up-regulate the number of activated NF-κB positive cells and stimulate I-κBα degradation. These results demonstrated that salusin-β could increase the expressions of inflammatory factors, at least in part, through increasing the activation of the I-κBα/NF-κB pathway, which might be one of the mechanisms for salusin-β to accelerate the progression of atherosclerosis. On the contrary, infusion of salusin-α into apoE-/- mice could not affect NF-κB signaling in the aorta, which indicated that atherosclerosis amelioration by salusin-α was not associated with NF-κB signaling.

However, there are some limitations about our study. Firstly, the point that salusin-α could reduce the plasma levels of IL-6 and TNF-α, without any effect on VCAM-1, MCP-1, NF-κB and I-κBα remains unclear. In fact, it has been reported that the mean concentration of serum salusin-β in healthy human serum is higher than that of salusin-α [22], [23]. Patients with atherosclerosis present up-regulated level of salusin-β but down-regulated level of salusin-α [23], [24]. In other words, the concentration of endogenous salusin-α is lower than salusin-β in the case of atherosclerosis. In light of the same doses of salusin-α and salusin-β used in the current study, the effect of a lower dose of salusin-α will be investigated in future researches. Secondly, we did not get the results of MCP-1 protein expression assayed by western blotting since the anti-MCP-1 antibody did not work well. In addition, due to the complication of atherosclerosis pathogenesis, other mechanisms may be involved in the promotion of atherosclerosis by salusin-β and the alleviation of atherosclerosis by salusin-α.

In summary, the present study found that exogenous salusin-β, but not salusin-α, could directly promote vascular inflammation in apoE-/- mice via the I-κBα/NF-κB pathway, resulting in the aggravation of atherosclerosis. These findings provide a further insight into the mechanisms of salusins in atherosclerosis, suggesting potential targets for the prevention and treatment of atherosclerosis.
